# Racial/Ethnic Differences in Electronic Cigarette Use and Reasons for Use among Current and Former Smokers: Findings from a Community-Based Sample

**DOI:** 10.3390/ijerph13101009

**Published:** 2016-10-14

**Authors:** Monica Webb Hooper, Stephanie K. Kolar

**Affiliations:** 1 Department of Psychology, College of Arts and Sciences, University of Miami, Coral Gables, FL 33146, USA; skolar@miami.edu; 2 Sylvester Comprehensive Cancer Center, Miami, FL 33136, USA

**Keywords:** electronic nicotine delivery systems, e-cigarette, smoking, racial/ethnic minorities, African Americans, Hispanics

## Abstract

The prevalence of e-cigarette use is increasing, yet few studies have focused on its use in racial/ethnic minority populations. We examined associations between race/ethnicity and e-cigarette use, plans to continue using e-cigarettes, and reasons for use among current/former smokers. Participants (285 in total; 29% non-Hispanic White, 42% African American/Black, and 29% Hispanic) were recruited between June and November 2014. Telephone-administered surveys assessed demographics, cigarette smoking, e-cigarette use, plans to continue using, and reasons for use. Analyses of covariance (ANCOVAs) and multivariable logistic regressions were conducted. African Americans/Blacks were significantly less likely to report ever-use compared to Whites and Hispanics (50% vs. 71% and 71%, respectively; *p* < 0.001). However, African American/Black ever users were more likely to report plans to continue using e-cigarettes compared to Whites and Hispanics (72% vs. 53% and 47%, respectively, *p* = 0.01). African American/Black participants were more likely to use e-cigarettes as a cessation aid compared to both Whites (*p* = 0.03) and Hispanics (*p* = 0.48). White participants were more likely to use e-cigarettes to save money compared to Hispanics (*p* = 0.02). In conclusion, racial/ethnic differences in e-cigarette use, intentions, and reasons for use emerged in our study. African American ever users may be particularly vulnerable to maintaining their use, particularly to try to quit smoking. These findings have implications for cigarette smoking and e-cigarette dual use, continued e-cigarette use, and potentially for smoking-related disparities.

## 1. Introduction

There has been a steep increase in the popularity of electronic nicotine delivery systems (ENDS), particularly electronic cigarettes (e-cigarettes), in the U.S. and internationally. E-cigarette use is associated with less exposure to toxicants relative to tobacco cigarettes [1,2], yet their contents include potential dangers such as carcinogens, volatile organic compounds [3,4], and heavy metals [5]. Increasing our understanding of e-cigarette use is important for public health. Knowledge regarding factors related to e-cigarette use has been derived largely from large population-based samples or internet-based convenience surveys. These studies have shown that current smoking is the most robust correlate of e-cigarette use among adults [6,7,8,9,10,11]. The prevalence of ever-using e-cigarettes among adult smokers ranges from 30% to 70% [7,12,13,14,15,16]. Among smokers in a state-level survey in Kansas, 14% of e-cigarette ever-users reported past-month use [12]. Thus, the prevalence of e-cigarette use is considerable and growing. However, remarkably little research has focused on e-cigarette use in racial/ethnic minority populations. Two such populations are African American and Hispanic smokers, for which smoking patterns, nicotine dependence, types of cigarettes smoked, and cessation rates differ from Whites [17,18].

The methodology of the extant research may limit our understanding of e-cigarette use in racial/ethnic minorities. That is, national or internet-based surveys that have reported e-cigarette use by demographic factors (e.g., King et al. [7,10]) have not oversampled racial/ethnic minority groups. Moreover, convenience samples recruited online may be less likely to include lower-income smokers who are also exposed to e-cigarette promotions. As such, rates of e-cigarette use in community-based versus population-based samples may differ. For instance, the prevalence estimates in community-based samples of hospitalized smokers [19] and substance abuse treatment seekers [20] were relatively high. As the science of racial/ethnic tobacco-related disparities continues to develop, it is imperative that we also begin to understand alternative tobacco product use and ENDS in racial/ethnic minority smokers. 

### 1.1. Reasons for E-Cigarette Use

Smokers report several reasons for using e-cigarettes. Among the most commonly cited reasons are smoking cessation [12,20,21,22], or reduction [20,23,24,25], and to lessen health risks [24,25]. Reasons for use also include enjoyment [22,25], curiosity [20,26], lower toxicity [21,22], and sensation seeking [8]. Smokers may also use e-cigarettes to circumvent smoking restrictions and to limit environmental tobacco smoke (ETS) exposure [20,21,25]. Previous research has also found that smokers who reported using e-cigarettes because they are cheaper, to reduce stress, or to quit smoking were more likely to decreases smoking and report an intention to quit [24]. This suggests that e-cigarette use among smokers may represent an opportunity to re-engage smokers in cessation efforts. However, growing evidence indicates that e-cigarette use does not promote smoking cessation [27,28], and may reduce the chances of quitting [29].

### 1.2. Racial/Ethnic Differences in E-Cigarette Use

Relatively few studies have examined racial/ethnic differences in e-cigarette use. To date, non-Hispanic Whites were more likely than racial/ethnic minorities to report e-cigarette use in samples of adults [6], young adults [13], and adolescents [30]. However, research among college students found no relationship with race/ethnicity [31]. Among current adult smokers, the evidence is limited and inconsistent. Some research has found no racial/ethnic differences in e-cigarette prevalence [7,10,32], while other studies have found lower rates in hospitalized African American [33] and Hispanic smokers relative to Whites [34]. Previous research has not compared the reasons for e-cigarette use by race/ethnicity. 

### 1.3. The Present Study

Racial/ethnic minorities have been underrepresented in the rapidly growing e-cigarette evidence base. E-cigarette use among smokers is increasing, often as a cessation aid. The purpose of the present study was to compare e-cigarette use (ever use, past 30 days use, and current use), plans to continue e-cigarette use, and reasons for use among African Americans, Hispanics, and non-Hispanic Whites. We focused on current adult smokers and former smokers because: (a) they may be particularly vulnerable to e-cigarette use and/or dual use; (b) few studies have considered racial/ethnic differences in e-cigarette use among former smokers; and (c) differences by race/ethnicity have implications for targeted intervention development. We conducted a cross-sectional, exploratory investigation in a community-based sample.

## 2. Materials and Methods

### 2.1. Participants and Data Collection

Participants were recruited in Florida through the internet, community outreach, flyers, churches, and healthcare settings from June to September 2014. We sought to recruit 300 participants with relatively equal representation of our three primary target groups, although there were no specific racial/ethnic inclusion criteria. Eligible participants were at least 18 years of age, able to speak English or Spanish, and were current (smoked at least 100 lifetime cigarettes and current daily or non-daily smoking) or former smokers (smoked at least 100 lifetime cigarettes, but no current smoking). Ineligible respondents included those who had another household member who participated previously, or who demonstrated cognitive impairment that hindered the study completion. Eligible participants provided verbal informed consent, followed by a 20 min telephone survey. Upon completion, participants received a $10 gift card. 

A total of 338 respondents were screened for eligibility. We excluded never-smokers (*n* = 9), those who were not interested (*n* = 9), and individuals who were unreachable for survey completion (*n* = 20). The current study included participants who were aware of e-cigarettes (*n* = 297), and identified as White (non-Hispanic), African American/Black, and Hispanic (any race) (final sample *N* = 285). All participants provided verbal informed consent. The University of Miami Institutional Review Board approved this study (ID: 20140247).

### 2.2. Measures

#### 2.2.1. Demographics and Smoking Status

Participants self-reported age, sex, marital status, annual household income, education, and smoking status (current or former smoker). Race/ethnicity was assessed using two items: (1) self-identification as Hispanic or Latino/a (yes or no); and (2) self-identification as Black (African American, Caribbean/Islander), White, Asian, Middle Eastern, Indian, Native American, Alaskan Native, Native Hawaiian or Other Pacific Islander, or Other/Multi-racial. The present study did not include the 12 individuals who self-identified as a category other than Black, White, or Hispanic (of any race).

#### 2.2.2. E-Cigarette Use

Single items assessed e-cigarette use and plans for use. Participants were asked, “Have you ever used an e-cigarette (yes or no)?” E-cigarette ever-users were asked “Have you used an e-cigarette at least once in the past 30 days (yes or no)?” “Do you now smoke e-cigarettes every day, some days, or not at all?” And, “Do you plan to continue e-cigarette use (yes or no)?” 

#### 2.2.3. Reasons for E-Cigarette Use

A 13-item measure was developed for the current survey guided by Etter & Bullen [21], who conducted an online survey and found that reasons for e-cigarette use included lower toxicity, dealing with cravings, withdrawal management, smoking cessation, their cheaper cost, and dealing with situations where smoking was prohibited. 

Items were rated on a 5-point Likert scale, ranging from (0) strongly disagree to (4) strongly agree (Table S1). The smoking cessation subscale assessed the use of e-cigarettes as a cessation aid (3 items). For example, “I use or used an e-cigarette to quit smoking.”. The nicotine withdrawal subscale assessed e-cigarette use to manage withdrawal symptoms (2 items). For instance, “I use or used an e-cigarette to deal with feeling bad after quitting (headaches, nausea, dizziness).” The harm reduction subscale assessed e-cigarette use to lessen exposure to the dangers of combustible cigarettes (3 items). For example, “I use or used an e-cigarette because it is less harmful than tobacco smoking.” The cost subscale assessed the use of e-cigarettes because combustible cigarettes are expensive (2 items). An item included, “I use or used an e-cigarette because it is/they are cheaper than smoking cigarettes.” The circumvent environmental tobacco smoke (ETS) policy/smoking bans subscale assessed e-cigarette use to evade policies against smoking in certain settings (e.g., indoor public places, playgrounds, hospital campuses; 3 items). For example, “I use or used an e-cigarette so I don’t have to go outside to smoke.” Total scores for each subscale were used in the analyses.

### 2.3. Statistical Analyses

Initial analyses included descriptive statistics for the overall sample and by racial/ethnic group. Racial/ethnic differences in demographics (age, sex, marital status, education, annual household income) and smoking status were evaluated using chi-squared tests for categorical variables and analyses of variance (ANOVAs) for continuous variables. Next, multivariable logistic regression analyses adjusting for all demographics and smoking status examined the independent associations between race/ethnicity and the odds of (1) ever use; (2) past 30 days use; and (3) plans to continue e-cigarette use. Analyses of covariance (ANCOVAs), adjusting for all demographics and smoking status, tested racial/ethnic differences in reasons for e-cigarette use among ever users. Given our a priori interest in racial/ethnic group differences, we compared groups pairwise. Analyses were conducted using IBM SPSS Statistics 23 and 24 (IBM Corporation, Armonk, NY, USA), and alpha was set at 0.05.

## 3. Results

### 3.1. Demographic Characteristics by Race/Ethnicity

As shown in Table 1, the overall sample was primarily female, single, lower-income, had completed ≥12 years of education, and were current smokers. Hispanics were significantly younger compared to White and African American/Black participants (*p* < 0.001). African American/Black participants were more likely than White and Hispanic participants to report an annual household income of less than $10,000 (chi-squared: 47.54; 6 df; *p* < 0.001), and the household incomes of Whites were most likely to exceed $40,000. African American/Black participants were more likely to report non-completion of high school compared to both White and Hispanic participants (chi-squared: 47.58; 6 df; *p* < 0.001), and White participants were most likely to complete college. African American/Black participants were more likely to be current (versus former) smokers, compared to both Hispanics and Whites (chi-squared: 9.23; 2 df; *p* = 0.01). The racial/ethnic differences in marital status and sex were not significant. 

### 3.2. Race/Ethnicity and E-Cigarette Use

Almost two-thirds of participants reported ever-use of e-cigarettes. As shown in Table 2, compared to Whites and Hispanics, African American/Black participants were the least likely to have ever used an e-cigarette (chi-squared: 13.63; 2 df; *p* < 0.001). There was no difference in ever-use between Whites and Hispanics. Among ever-users, almost one-half reported past 30 days e-cigarette use, with no differences by race/ethnicity. Over one-third of ever users reported current e-cigarette use on some days or every day, again with no racial/ethnic differences. However, there was a significant difference in behavioral intentions; a greater proportion of African American/Black ever-users reported plans to continue using e-cigarettes compared to Whites and Hispanics (chi-squared: 8.69; 2 df; *p* < 0.001).

Multivariable analyses examined the independent associations between race/ethnicity and e-cigarette use and intentions to continue use. After controlling for demographics and smoking status, African American/Black participants were less likely to have ever used an e-cigarette compared to Whites (Table 3). Ever use was also independently associated with age, such that older participants were less likely to have ever used an e-cigarette. Among ever-users, race/ethnicity was not associated with past 30 days use. However, after adjusting for covariates, race/ethnicity was related to plans to continue using e-cigarettes. Compared to Whites, African American/Black participants were significantly more likely to endorse plans to continue e-cigarette use (adjusted odds ratio (AOR) = 2.56, 95% confidence interval (CI): 1.03–6.37). The difference between Hispanic and White ever users was not significant. Finally, smoking status was independently associated with plans to continue e-cigarette use. Among ever users, males (vs. females) were significantly less likely to report plans to continue e-cigarette use (AOR = 0.45, 95% CI: 0.22–0.91). Finally, current smokers were over three times more likely to report plans to continue using e-cigarettes relative to former smokers (AOR = 3.24, 95% CI: 1.26–8.33).

### 3.3. Race/Ethnicity and Reasons for E-Cigarette Use

Multivariable analyses controlling for demographics and smoking status tested racial/ethnic differences in reasons for using e-cigarettes in ever-users (Table 4). We were interested in comparing groups pairwise. We found significant group differences in using e-cigarettes for smoking cessation. Specifically, African American/Black participants were more likely to use e-cigarettes as a cessation aid compared to both Whites (*p* = 0.03) and Hispanics (*p* = 0.48). There was also a significant difference in the cost subscale. White participants were more likely to use e-cigarettes to reduce smoking-related financial costs compared to Hispanics (*p* = 0.02), but not African Americans/Blacks. There were no differences in e-cigarette use for nicotine withdrawal management, harm reduction, or to circumvent ETS policies or bans (all *p* > 0.05).

## 4. Discussion

This study was among the first to test racial/ethnic differences in e-cigarette use and reasons for use among individuals with a tobacco smoking history. This study contributes to the literature by specifically focusing on the experience of racial/ethnic minorities and by seeking a community-based sample. Thus, an underlying goal of the study was to examine these questions among those who may be underrepresented in national or internet surveys. Across all metrics, the prevalence of e-cigarette use was high in this sample. Approximately half of participants reported using e-cigarettes within the past 30 days, and over one-third reported at least non-daily use. In addition, most ever-users reported intentions to continue e-cigarette use. With regard to racial/ethnic differences, African Americans were less likely to have ever tried an e-cigarette compared to Whites. Yet, African American ever-users were more likely to plan to maintain their e-cigarette use. Relative to both Whites and Hispanics, African Americans were also more interested in using e-cigarettes to quit smoking. Whites were more likely to use e-cigarettes to save money, in comparison to Hispanics. These findings confirm previous research showing a high prevalence of e-cigarette use among smokers [7,12,13,14,15,16], and highlight sociodemographic differences that might influence future e-cigarette use trends. 

Consistent with previous research [8,12], the prevalence of e-cigarette ever-use was high in the current sample. This could reflect the growing use of these products, the sampling method, and/or the strong interest in trying e-cigarettes in underrepresented populations. We did not find racial/ethnic differences on indicators of current e-cigarette use. However, current smokers were more likely to have tried an e-cigarette compared to former smokers. This is consistent with several previous studies that also identified current smokers as the most susceptible group to ever-use e-cigarettes (e.g., King et al. [10]). Data are limited on the prevalence of e-cigarette use among racial/ethnic minorities. Some studies have included analyses by race/ethnicity, but aggregated the data for racial minorities [13], and/or did not report prevalence rates among tobacco smokers [6]. Among smokers, some research has found no racial/ethnic differences in e-cigarette prevalence [7,10,32], while another study found lower rates in hospitalized African American and Hispanic smokers relative to Whites [34]. 

We found that most e-cigarette ever-users in this sample endorsed plans to continue use. Current smokers were more likely to report plans to continue using e-cigarettes compared to former smokers. Although the present study cannot provide a definitive interpretation, the findings have implications for dual use. It could be that former smokers were less interested in continuing to use e-cigarettes because they have quit successfully. In contrast, current smokers may plan to maintain e-cigarette use due to pleasant initial experiences, or to reduce health risks, circumvent ETS policies, or in preparation for quitting smoking. There were also independent associations between sex and race with intentions to continue e-cigarette use. Females and African Americans were more likely to report plans to continue e-cigarette use relative to their counterparts. One possible explanation is that because these groups tend to have greater difficulty in quitting smoking [35,36], they may intend to use e-cigarettes as a cessation aid. With the recent introduction of mentholated e-cigarette flavoring, females and African Americans are likely targets for e-cigarette marketing [37]. These findings may signal a vulnerability to e-cigarettes and have implications for a possible increase in prevalence in these groups. Future research is needed to examine these possibilities.

Smoking cessation is among the most common reasons for e-cigarette use [12,20,21,22]. To our knowledge, no previous research has compared this reason by race/ethnicity. In the current sample, we found that African American/Black participants were more likely to use e-cigarettes as a cessation aid compared to both Whites and Hispanics. This finding has important implications for quitting smoking and for dual use. Research has documented that African Americans are less likely to quit smoking compared to Whites [17]. African Americans in the current sample were also less likely to be former smokers. There are multiple contributory factors to this disparity, but at the individual-level, African Americans may have lower expectancies for smoking cessation treatments and may underestimate the difficulty of quitting [38]. This may, in part, explain the lower likelihood of utilizing evidence-based cessation treatments, including pharmacotherapy, compared to Whites [39]. In contrast, smokers may perceive greater benefits from e-cigarettes versus nicotine replacement therapy [40]. To the extent that African Americans use e-cigarettes as a cessation aid, the use of evidence-based pharmacotherapy may further decline. The evidence regarding e-cigarette use and smoking cessation is equivocal; thus, the impact of this race difference is unknown. It is possible that African Americans’ e-cigarette use as a smoking cessation aid could either widen disparities in quitting [12,28,29], or promote pharmacotherapy use [23] and facilitate quitting [15]. Future research is needed to examine the impact of dual tobacco smoking and e-cigarette use on disparities in smoking cessation.

Research has shown that cost is a reason for e-cigarette use among smokers. That is, e-cigarettes are perceived to be less expensive than tobacco smoking [40,41]. In the current sample, we found that White ever users were more likely than Hispanics to endorse saving money as a reason for e-cigarette use. While cost is a practical reason, e-cigarette use may not result in financial savings if used simultaneously with tobacco cigarettes. Indeed, Grace et al. [42] found that e-cigarettes have the potential to reduce the demand for tobacco cigarettes, yet may foster dual use. Finally, exposure to e-cigarette advertisements claiming lower costs compared to tobacco cigarettes may influence interest in trying e-cigarettes [43]; thus, future research should examine racial/ethnic differences in advertisement exposure and subsequent interest and behavior. 

This study has several strengths and limitations. The a priori foci on current and former smokers, and the testing of racial/ethnic differences resulted in a sample with substantially greater diversity than other e-cigarette studies. We also sought to recruit smokers who may be underrepresented in national surveys or internet panels (e.g., low socioeconomic status). Importantly, we controlled for income and education to test the independent association between race/ethnicity and e-cigarette use. Limitations included a convenience sample that may limit the generalizability of the findings, the restricted number of items surveyed, and the cross-sectional design. Also, we did not assess whether the former smokers had a history of regular smoking, or the timing of first e-cigarette use. Given that this is the first study (to our knowledge) to test these relationships with a focus on race/ethnicity, findings are considered exploratory and warrant replication.

## 5. Conclusions

With the rapid increase in e-cigarette use in the U.S., it is important to understand their use across communities. This study found more similarities than differences among Whites, African Americans/Blacks, and Hispanics with a history of tobacco smoking. However, the observed racial/ethnic differences have implications for the prevalence of e-cigarette use and dual use. African American/Black ever-users expressed the strongest intentions to continue use, and for smoking cessation purposes. If e-cigarette use truly lowers the chances of cessation, this could have a negative impact on the health of African American/Black smokers and widen disparities in quitting. Smokers and former smokers may benefit from education on the state-of-the-scientific evidence regarding e-cigarette cost, and their use as a smoking cessation strategy. 

## Figures and Tables

**Table 1:** Demographic characteristics by racial/ethnic group.

		Racial/Ethnic Group			
	Total	White	African American/Black	Hispanic	
	(*N* = 285)	(*n* = 82)	(*n* = 119)	(*n* = 84)	*p* Value
**Age (Mean, Standard Deviation)**	42.8 (12.9)	44.4 (13.3)	45.1 (11.7)	38.2 (13.0)	<0.001
**% (*n*)**					
**Sex**					0.56
Female	61% (173)	62% (51)	57% (68)	64% (54)	
Male	39% (112)	38% (31)	43% (51)	36% (30)	
**Marital status**					0.05
Unmarried/Single	56% (160)	45% (37)	64% (76)	56% (47)	
Married/Living with a partner	20% (56)	28% (23)	13% (15)	21% (18)	
Separated/Divorced/Widowed	24% (69)	27% (22)	23% (28)	23% (19)	
**Annual household income**					<0.001
Under $10,000	36% (102)	15% (12)	52% (62)	34% (28)	
$10,001–$20,000	19% (54)	15% (12)	22% (26)	19% (16)	
$21,001–$40,000	26% (73)	34% (27)	20% (23)	28% (23)	
$40,001 or more	19% (52)	36% (29)	6% (7)	19% (16)	
**Education**					<0.001
Less than HS diploma/GED	20% (58)	5% (4)	35% (42)	14% (12)	
HS diploma/GED	24% (68)	19% (16)	29% (34)	22% (18)	
Business/Technical training or some college	36% (103)	43% (35)	29% (35)	39% (33)	
College degree (2-year, 4-year, or graduate)	20% (56)	33% (27)	7% (8)	25% (21)	
**Smoking Status**					0.01
Current Cigarette Smoker	77% (219)	72% (59)	86% (102)	69% (58)	
Former Cigarette Smoker	23% (66)	28% (23)	14% (17)	31% (26)	
**Has heard about e-cigarettes**	99% (285)	100% (82)	98% (119)	99% (84)	0.52

HS: High School; GED: Graduate Equivalency Degree.

**Table 2:** Unadjusted Analyses of E-Cigarette Use by Racial/Ethnic Group.

		Racial/Ethnic Group			
	Total	White	African American/Black	Hispanic	
	(*N* = 285)	(*n* = 82)	(*n* = 119)	(*n* = 84)	*p* Value
**Ever used an e-cigarette (Yes)**	62% (177)	71% (58) ^a^	50% (59) ^b^	71% (60) ^a^	<0.001
**Ever Users:**	*n* = 177	*n* = 58	*n* = 59	*n* = 60	
Used an e-cigarette in the past 30 days (Yes) *	49% (86)	52% (30) ^a^	49% (29) ^a^	45% (27) ^a^	0.76
**Currently smoke e-cigarettes **					0.51
Not at all	62% (109)	67% (39) ^a^	53% (31) ^a^	65% (39) ^a^	
Some Days/Everyday	38% (68)	33% (19) ^a^	48% (28) ^a^	35% (21) ^a^	
Plans to keep using e-cigarettes *	57% (100)	53% (30) ^a^	72% (42) ^b^	47% (28) ^a^	0.01

* = among ever-users, *n* = 177; ^a^ indicates that there is not a significant difference; ^b^ indicates statistically significant difference from the other racial/ethnic groups.

**Table 3:** Multivariable Logistic Regressions of E-Cigarette Use and Plans to Continue Use.

		Ever Use ^1^		Past 30 Days Use ^2^		Plans to Continue ^2^	
		AOR	(95% CI)	AOR	(95% CI)	AOR	(95% CI)
**Characteristic**							
**Age**		0.95	(0.93–0.97)	1.02	(0.99–1.04)	1.01	(0.98–1.04)
**Sex**							
	Female		Reference		Reference		Reference
	Male	1.21	(0.68–2.17)	1.05	(0.54–2.02)	0.45	(0.22–0.91)
**Marital Status**							
	Unmarried/Single		Reference		Reference		Reference
	Married/Living with a partner	0.85	(0.39–1.84)	1.21	(0.52–2.86)	1.65	(0.65–4.15)
	Separated/Divorced/Widowed	1.03	(0.51–2.09)	0.77	(0.32–1.84)	0.84	(0.32–2.19)
**Annual Household Income**							
	Under $10,000		Reference		Reference		Reference
	$10,001–$20,000	0.66	(0.31–1.40)	1.06	(0.40–2.80)	1.38	(0.47–4.08)
	$21,001–$40,000	1.30	(0.59–2.85)	2.03	(0.84–4.93)	0.65	(0.25–1.67)
	$40,001 or more	1.28	(0.48–3.39)	2.08	(0.76–5.75)	0.40	(0.14–1.18)
**Education**							
	Less than HS diploma/GED		Reference		Reference		Reference
	HS diploma/GED	1.67	(0.75–3.70)	0.56	(0.20–1.54)	0.98	(0.33–2.93)
	Technical training or some college	1.67	(0.76–3.71)	0.64	(0.24–1.74)	1.94	(0.65–5.79)
	College degree	2.74	(0.97–7.71)	0.40	(0.12–1.31)	1.28	(0.36–4.55)
**Smoking Status**							
	Former Smoker		Reference		Reference		Reference
	Current Smoker	3.70	(1.86–7.33)	1.26	(0.53–2.99)	3.21	(1.25–8.28)
**Race/Ethnicity**							
	White		Reference		Reference		Reference
	African American/Black	0.46	(0.22–0.98)	1.02	(0.45–2.33)	2.50	(1.02–5.91)
	Hispanic (any race)	0.95	(0.43–2.09)	1.04	(0.47–2.32)	0.81	(0.34–1.80)

^1^ *n* = 285; ^2^ *n* = 177, ever users only; Analyses were controlled for all demographics and smoking status; Adjusted Odds Ratio (AOR) of less than 1.0 indicates decreased odds of e-cigarette use; CI: Confidence Interval; “Reference” indicates the reference group for categorical variables.

**Table 4:** Reasons for E-Cigarette Use among Ever-Users by Racial/Ethnic Group.

		Racial/Ethnic Group		
	Total	White	African American/Black	Hispanic
	(*N* = 177)	(*n* = 58)	(*n* = 59)	(*n* = 60)
	M (SD)	M (SD)	M (SD)	M (SD)
**Smoking Cessation (range = 0–12)**	7.97 (3.6)	7.50 (3.6) ^a^	8.81 (3.2) ^b^	7.56 (3.9) ^a^
**Nicotine Withdrawal (range = 0–8)**	4.39 (2.1)	4.54 (2.1) ^a^	4.58 (2.1) ^a^	4.05 (2.1) ^a^
**Harm Reduction (range = 0–12)**	7.79 (2.8)	7.96 (2.5) ^a^	7.85 (2.8) ^a^	7.56 (3.0) ^a^
**Cost (range = 0–8)**	4.15 (2.5)	4.59 (2.6) ^a^	4.34 (2.5) ^a^	3.54 (2.4) ^b^
**Circumvent ETS Policy/Bans (range = 0–12)**	7.58 (2.9)	7.84 (3.2) ^a^	7.39 (3.1) ^a^	7.53 (2.5) ^a^

^a^ indicates that there is not a significant difference; ^b^ indicates statistically significant differences from the other racial/ethnic groups, *p* < 0.05; Analyses adjusted for all demographics and smoking status; Higher values indicate greater agreement with the reason for e-cigarette ever-use; ETS: Environmental Tobacco Smoke; M: Mean; SD: Standard Deviation.

## Supplementary Materials

The following are available online at www.mdpi.com/1660-4601/13/10/1009/s1, Table S1: Reasons for E-Cigarette Use Scale.
