# Supplementary material for: Racial/Ethnic Differences in Electronic Cigarette Use and Reasons for Use among Current and Former Smokers: Findings from a Community-Based Sample
Source: Int J Environ Res Public Health. 2016 Oct 14;13(10):1009. doi: 10.3390/ijerph13101009 (PMC5086748; doi:10.3390/ijerph13101009)
Supplement: Supplementary file 1 [file ijerph-13-01009-s001.pdf]

Supplementary Materials: Racial/Ethnic Differences in Electronic Cigarette Use and Reasons for Use among Current and Former Smokers: Findings from a Community-Based Sample

Monica Webb Hooper and Stephanie K. Kolar

Table S1. Reasons for E-Cigarette Use Scale.

	**Strongly Disagree**	**Disagree**	**Neutral**	**Agree**	**Strongly Agree**
Because it is less harmful than tobacco	☐	☐	☐	☐	☐
To deal with cravings for tobacco	☐	☐	☐	☐	☐
To quit smoking	☐	☐	☐	☐	☐
To avoid smoking traditional cigarettes again after quitting	☐	☐	☐	☐	☐
To deal with feeling bad after quitting (headaches, nausea, dizziness)	☐	☐	☐	☐	☐
Because it is cheaper than smoking traditional cigarettes	☐	☐	☐	☐	☐
To avoid bothering others with tobacco smoke	☐	☐	☐	☐	☐
To deal with times where I couldn't smoke traditional cigarettes because of a smoking ban (at work, in public places, etc.)	☐	☐	☐	☐	☐
So I don't have to go outside to smoke	☐	☐	☐	☐	☐
To prepare for quitting smoking traditional cigarettes	☐	☐	☐	☐	☐
To cut down using traditional cigarettes, but I don't want to quit smoking	☐	☐	☐	☐	☐
Because I will save money	☐	☐	☐	☐	☐
Just because (e.g., I can do it anywhere)	☐	☐	☐	☐	☐
